# Mapping a quantitative trait locus for resistance to bacterial grain rot in rice

**DOI:** 10.1186/1939-8433-6-13

**Published:** 2013-05-21

**Authors:** Ritsuko Mizobuchi, Hiroyuki Sato, Shuichi Fukuoka, Takanari Tanabata, Seiya Tsushima, Tokio Imbe, Masahiro Yano

**Affiliations:** National Institute of Agrobiological Sciences, 2-1-2 Kannondai, Tsukuba, Ibaraki 305-8602 Japan; National Agriculture and Food Research Organization (NARO), Institute of Crop Science, 2-1-18 Kannondai, Tsukuba, Ibaraki 305-8518 Japan; RIKEN Center for Sustainable Resource Science, Gene Discovery Research Group, Tsukuba, Ibaraki 305-0074 Japan; National Institute of Agro-Environmental Sciences, 3-1-3 Kannondai, Ibaraki 305-8604 Japan; National Agricultural and Food Research Organization, 3-1-1 Kannondai, Tsukuba, Ibaraki 305-8517 Japan; National Institute of Agrobiological Sciences, 1-2 Ohwashi, Tsukuba, Ibaraki 305-8634 Japan

**Keywords:** *Oryza sativa* L, Disease resistance, Bacterial grain rot, *Burkholderia glumae*, QTL, Cut-panicle inoculation, Mapping

## Abstract

**Background:**

Bacterial grain rot (BGR), caused by the bacterial pathogen *Burkholderia glumae*, is a destructive disease of rice. Because BGR tends to be highly affected by environmental conditions such as temperature and humidity, it is difficult to evaluate BGR resistance of diverse cultivars with different heading dates by using field inoculation. Molecular tagging of genes involved in BGR is an important objective for rice breeding.

**Results:**

In this study, we mapped a quantitative trait locus (QTL) for BGR resistance by a modified cut-panicle inoculation method. First, we assessed the levels of BGR resistance in 84 cultivars by a standard cut-panicle inoculation technique, in which panicles are harvested and inoculated in the laboratory under controlled conditions. For the genetic analysis, we selected two cultivars: Kele, a resistant traditional lowland cultivar (*indica*) that originated in India, and Hitomebore, a susceptible modern lowland cultivar (*temperate japonica*) from Japan. Second, by comparing the susceptibility of Kele and Hitomebore spikelets before and up to 3 days after anthesis, we found a dramatic decline in susceptibility at 1 day after anthesis in Kele but not in Hitomebore. Thus, we applied a modified method by inoculating spikelets at 1 day after anthesis for further analysis. To search for QTLs associated with BGR resistance, we measured the ratio of diseased spikelets (RDS, an index reflecting both quantity and severity of infection) and the ratio of diseased spikelet area (RDSA) in 110 backcrossed inbred lines (BILs) derived from a cross between Kele and Hitomebore. One major QTL associated with both RDS and RDSA was detected on the long arm of chromosome 1. This QTL explained 25.7% and 12.1% of the total phenotypic variance in RDS and RDSA in the BILs, respectively, and the Kele allele increased BGR resistance.

**Conclusions:**

We mapped a major QTL for BGR resistance on the long arm of chromosome 1. These results clearly demonstrated that genetic analysis of BGR resistance in rice can be effectively performed and that this trait could be a target of marker-assisted selection in rice breeding programs.

**Electronic supplementary material:**

The online version of this article (doi:10.1186/1939-8433-6-13) contains supplementary material, which is available to authorized users.

## Background

*Burkholderia glumae* causes bacterial grain rot (BGR) and seedling rot in rice (*Oryza sativa* L.), which are increasingly important diseases in global rice production (Ham et al. 2011b). Since *B. glumae* was first discovered in Japan (Goto and Ohata 1956; Goto et al. 1^1987^; Kurita and Tabei 1967; Uematsu et al. 1976), it has also been reported in other countries in East Asia (Chien and Chang 1987; Cottyn et al. 1996a; Cottyn et al. 1996b; Jeong et al. 2003; Luo et al. 2007; Trung et al. 1993) and Latin America (Nandakumar et al. 2007b; Zeigler and Alvarez 1989). In the USA, *B. glumae* has been identified as the major causal agent of BGR (Nandakumar et al. 2005; Nandakumar et al. 2009; Shahjahan et al. 2000). In the southern United States, yield losses caused by outbreaks of BGR in rice fields in Louisiana were as much as 40% in 1995 and 1998; significant losses caused by this disease were also experienced in more recent years (Ham et al. 2011a; Ham et al. 2011b; Nandakumar et al. 2009; Shahjahan et al. 2000; Zhou et al. 2011). BGR in rice occurs by both primary and secondary infection (Tsushima 1996; Tsushima et al. 1991; Tsushima et al. 1996). Primary infection occurs when seeds contaminated with *B. glumae* are sown and transplanted into fields, and seedling rot appears in some infected plants. At heading (anthesis), plants that are located near the diseased primary-infected plants are also attacked by the pathogen, thus establishing secondary infection. After infection, the color of the spikelets changes from the normal green color to reddish brown. Eventually, the infection may cause unfilled or aborted grains (Ham et al. 2011b).

The severity of BGR infection is affected by several endogenous and exogenous factors, such as host susceptibility, inoculum density, humidity, and temperature conditions (Tsushima 1996). The susceptibility of spikelets changes with time during a critical period from shortly before to shortly after flowering (Tsushima et al. 1995), so the occurrence of BGR is highly affected by environmental conditions around the heading date (Tsushima 1996; Tsushima et al. 1985). High humidity at the time of heading is conductive to the infection of the spikelets (Tsushima et al. 1995). Because the optimal temperature range for the growth of *B. glumae* is relatively high (30–35°C) (Kurita et al. 1964; Tsushima et al. 1986), this disease occurs primarily in tropical and semi-tropical countries. Global warming may cause BGR to become even more destructive (Ham et al. 2011b).

Many studies have been performed to understand the genetic control of BGR resistance. Although no source of complete resistance has been discovered (Miyagawa and Kimura 1989; Shahjahan et al. 2000), several cultivars show lower disease severity than others and appear to be partially resistant to BGR (Goto and Watanabe 1975; Groth et al. 2007; Imbe et al. 1986; Mogi and Tsushima 1985; Nandakumar et al. 2007a; Nandakumar and Rush 2008; Pinson et al. 2010; Prabhu and Bedendo 1988; Sayler et al. 2006; Sha et al. 2006; Takita et al. 1988; Wasano and Okuda 1994; Yasunaga et al. 2002). These resistant cultivars were evaluated in fields by spray inoculation at the heading stage or syringe inoculation at the booting stage. However, because BGR tends to be highly affected by environmental conditions such as humidity and temperature, it is difficult to evaluate BGR resistance of cultivars with different heading dates by using field inoculation (Tsushima 1996). To minimize environmental variation at the time of inoculation, the ‘cut-panicle inoculation’ method was created (Miyagawa and Kimura 1989). This method entails the collection of panicles from field-grown plants and their inoculation under controlled conditions at the time of flowering. Because the correlation coefficient between the disease rating obtained by cut-panicle inoculation and that obtained by pot inoculation is very high (*r* = 0.868), cut-panicle inoculation is recognized as a useful method for evaluating BGR resistance (Miyagawa and Kimura 1989).

Because of the difficulty in reliable evaluation of BGR resistance in rice, only one QTL analysis of BGR resistance has been published (Pinson et al. 2010). The authors detected one major QTL on chromosome 3 and 11 minor QTLs in a set of recombinant inbred lines developed from crosses between Lemont (susceptible) and TeQing (resistant). Disease was scored following spray inoculation in the field. The authors also assessed flowering time and identified a major QTL on chromosome 3, near the major QTL for BGR resistance, associated with flowering. Panicles of late-flowering plants experience cooler temperatures (which are less conductive to disease development during grain fill) than do those of earlier-flowering plants. Therefore, it is possible that the disease resistance mapped to chromosome 3 is a pleiotropic effect of the major QTL for heading date (Pinson et al. 2010).

To elucidate the genetic control of BGR resistance in rice, we conducted QTL analysis of BGR using backcrossed inbred lines (BILs) derived from a cross between Kele (resistant) and Hitomebore (susceptible). To minimize the effect of heading date on BGR and to obtain the greatest degree of discrimination between susceptible and resistant plants, we employed a modified assessment method for BGR resistance during the genetic analysis. By using this method, we successfully mapped a major BGR-resistance QTL on chromosome 1.

## Results

### Screening cultivars for BGR resistance by cut-panicle inoculation

**Figure 1:**
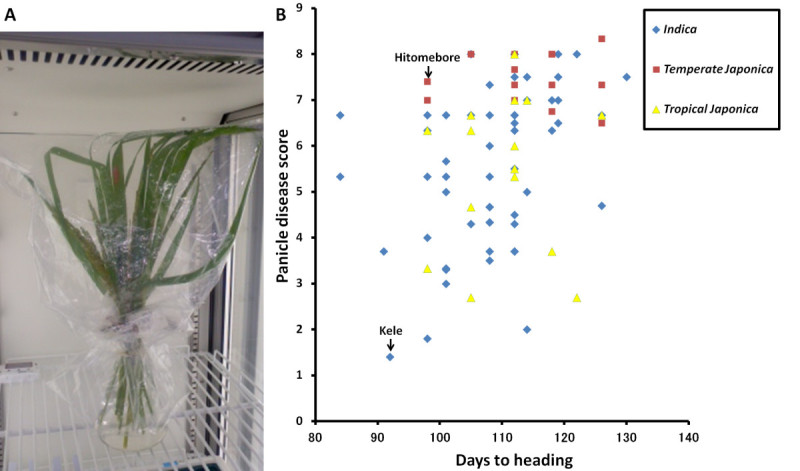
**Screening cultivars for BGR resistance by cut-panicle inoculation.** (**A**) Cut-panicle inoculation. Inoculated panicles were placed in an Erlenmeyer flask, wrapped in a transparent plastic bag, and placed in a growth chamber (see Methods). Panicles were scored for disease 6 days after inoculation. (**B**) Relationship between panicle disease score and heading date for cultivars evaluated in 2010. Blue diamonds, red squares, and yellow triangles indicate *indica*, *temperate japonica*, and *tropical japonica* cultivars, respectively.

We measured the levels of BGR resistance of 84 rice cultivars by the standard cut-panicle inoculation method (Figure 1A). These cultivars included 62 accessions from WRC [the World Rice Collection of the National Institute of Agrobiological Sciences (NIAS)]. Nine of the cultivars were previously reported to be resistant to BGR (Prabhu and Bedendo 1988; Wasano and Okuda 1994). The set consisted of 55 cultivars of *indica*, 15 cultivars of *tropical japonica*, and 14 cultivars of *temperate japonica* (Additional file 1: Table S1). The distribution of panicle disease scores of the cultivars evaluated in 2010 is shown in Figure 1B. The disease scores ranged from 1.4 to 8.0 (*indica*), from 2.7 to 8.0 (*tropical japonica*), and from 6.5 to 8.3 (*temperate japonica*), with Kele (*indica*) and Nishihomare (*temperate japonica*) being the most resistant and the most susceptible cultivars, respectively (Additional file 1: Table S1, Figure 1B). These results indicate wide variation in BGR resistance among the *indica* and *tropical japonica* cultivars, whereas all of the *temperate japonica* cultivars were moderately to highly susceptible.

**Figure 2:**
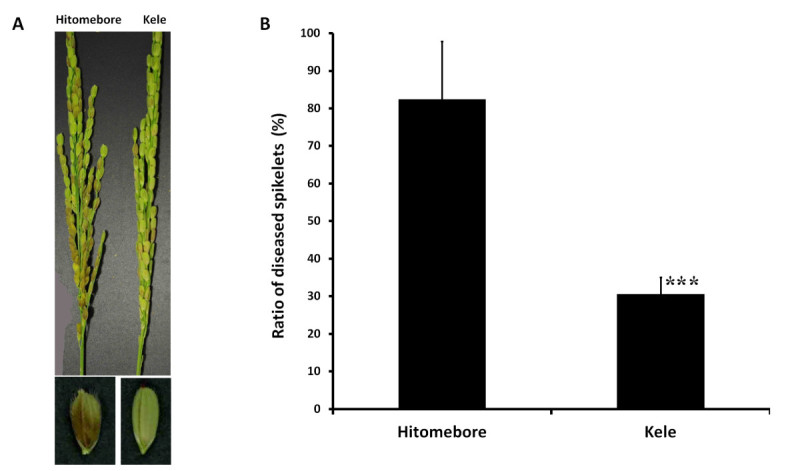
**BGR resistance of parental cultivars selected for QTL analysis.** (**A**) Differences in BGR resistance between the susceptible cultivar, Hitomebore (left), and the resistant cultivar, Kele (right), measured by standard cut-panicle inoculation. (**B**) Comparison of BGR resistance between Hitomebore and Kele by standard cut-panicle inoculation. Error bars indicate S.D. ***P < 0.0001 (Student’s *t* test).

To minimize the effect of differences in heading date on BGR resistance in further analysis, we selected two cultivars with almost the same heading date, Kele (the most resistant; headed in 92 days) and Hitomebore (much more susceptible; disease score of 7.4; headed in 98 days) (Figure 1B). Hitomebore is a modern elite lowland cultivar (*temperate japonica*) from Japan with good eating quality, and it is widely grown in Japan. Kele is a traditional lowland cultivar that originated in India. Six days after cut-panicle inoculation, many spikelets of Hitomebore had brown lesions; in contrast, few spikelets of Kele had brown lesions, and most spikelets had no lesions at all (Figure 2A). Therefore, we found that we could score BGR resistance 6 days after inoculation. To confirm the BGR resistance of Kele and Hitomebore, we performed cut-panicle inoculation and pot inoculation and measured the ratio of diseased spikelets (RDS), an index that considers both quantity and severity of infection (Figure 2B and Additional file 2: Table S2). The means and standard errors of RDS of Hitomebore and Kele by cut-panicle inoculation were 82.4 ± 15.4% and 30.6 ± 4.4%, respectively, and the difference was highly significant (*P* < 0.0001). Although the scores of pot inoculation were lower than those of cut-panicle inoculation, the means and standard errors of RDS of Hitomebore and Kele by pot inoculation were 50.4 ± 12.3% and 26.0 ± 12.2%, respectively, and the difference was significant (*P* < 0.01). During maturation, RDS of Kele of 20 days after pot inoculation remained lower than that of Hitomebore, and the grain sterility rate of Kele of 30 days after pot inoculation was lower than that of Hitomebore (Additional file 2: Table S2). Therefore, we confirmed that Kele was highly resistant and Hitomebore was highly susceptible in two different assays.

### Modification of cut-panicle inoculation method

**Figure 3:**
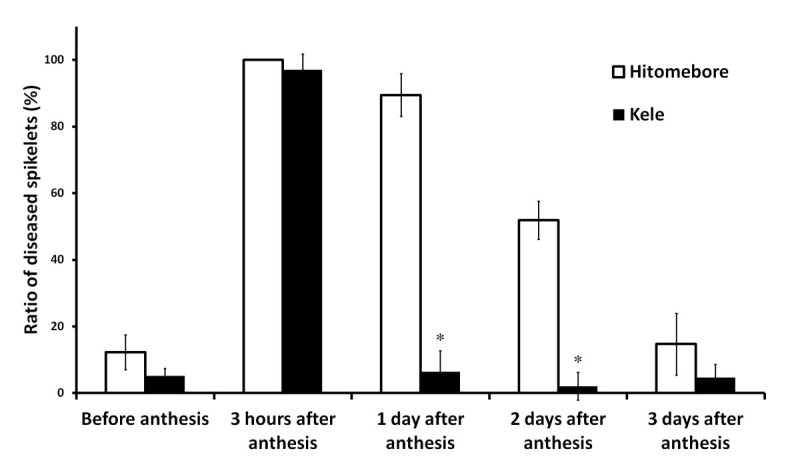
**Susceptibility to BGR of spikelets as a function of heading stage, measured by cut-panicle inoculation.** White bars indicate Hitomebore and black bars indicate Kele; error bars indicate S.D. *P < 0.05 (Student’s *t* test) between Hitomebore and Kele at each time point.

In the standard cut-panicle inoculation method, the panicles are inoculated 3 days after the first spikelets undergo anthesis. Because spikelets within the same panicle are not synchronized on the day of inoculation, the panicles contain spikelets before anthesis and spikelets 0, 1, 2, and 3 days after anthesis. We measured RDS of Kele and Hitomebore by inoculating panicles containing only spikelets before anthesis or spikelets at 0, 1, 2, or 3 days after anthesis (Figure 3). RDS of Hitomebore was 100% for spikelets inoculated within 3 hours after anthesis, decreased but remained relatively high for plants inoculated 1 and 2 days after anthesis, and then decreased dramatically for plants inoculated 3 days after anthesis. Spikelets inoculated before anthesis were resistant, indicating that Hitomebore had almost the same developmental pattern of BGR resistance as those of cultivars reported previously. In contrast, Kele showed a very different pattern: although it had the same high RDS as Hitomebore when spikelets were inoculated within 3 hours after anthesis, the RDS of Kele inoculated one day after anthesis was considerably lower (5.6 ± 6.3%) than that of Hitomebore (89.5 ± 6.5%) (*P* < 0.05). Therefore, we concluded that inoculating panicles containing only spikelets at 1 day after anthesis (hereafter, ‘modified cut-panicle inoculation’) would improve the sensitivity of BGR screening.

### Detection of a QTL for resistance to BGR by the modified cut-panicle inoculation method

**Figure 4:**
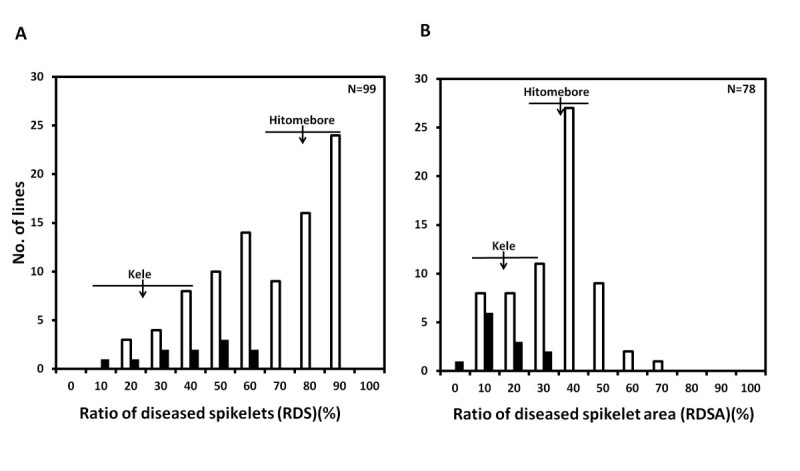
**BGR resistance of backcrossed inbred lines (BILs) derived from a cross between Kele and Hitomebore.** Frequency distributions of (**A**) the ratio of diseased spikelets (RDS) and (**B**) the ratio of diseased spikelet area (RDSA) assessed by the modified method of cut-panicle inoculation, classified by the genotype for marker P0684. White and black bars represent lines homozygous for the Hitomebore and Kele alleles, respectively. One line was found to be heterozygous at P0684 and is not shown. Arrows indicate the mean values of Kele and Hitomebore. Horizontal lines under the arrows indicate the standard deviations.

For QTL analysis, we measured the ratio of diseased spikelet area (RDSA) as well as RDS. The inoculated spikelets were imaged by using a scanner and RDSA was calculated by SmilePlant (Software of Measuring Image for Lesion Estimation), which we developed for this analysis. One drawback to this method was that in Kele, the spikelet color changes from green to deep purple during maturation (biological color change). This characteristic was also seen in some of the BILs. In most cases, we could measure RDS and RDSA until 8 days after inoculation, because the biological color change had not yet begun. However, in some cases, the biological color change starts just after heading when the plant is exposed to environmental conditions such as strong winds from a typhoon. In this study, once any biological color change had occurred, we could no longer measure RDSA. As a result, we could acquire data for RDS from 100 lines and for RDSA from 79 lines. The BILs showed a wide range of variation for both RDS (11.9% to 100%), and RDSA (5.5% to 71.0%) (Figure 4). The BILs also showed wide ranges of variation for other agronomic traits (Additional file 3: Figure S1), and correlation coefficients were calculated between BGR resistance (RDS and RDSA) and agronomic traits in the BILs (Table 1). The correlation between RDS and RDSA was significant at the 1% level (*r* = 0.790). Culm length, panicle length, number of panicles, spikelet length, and spikelet width were not significantly correlated with either RDS or RDSA. The correlation between heading date and RDSA was negative and significant at the 5% level (*r* = −0.247), but the correlation between heading date and RDS was not significant.

**Table 1:** **Correlation coefficients between BGR resistance (RDS and RDSA) and agronomic traits in BILs derived from a cross between Kele and Hitomebore** ^**a**^

	Ratio of diseased spikelets (RDS)^b^	Ratio of diseased spikelet area (RDSA)^b^
Ratio of diseased spikelet area (RDSA)	0.790**	
Culm length	−0.039	0.067
Panicle length	0.004	−0.096
Number of panicles	0.022	0.044
Spikelet length^c^	0.168	0.153
Spikelet width^c^	−0.077	0.006
Heading date	−0.113	−0.247*

^a^ BGR resistance and agronomic traits were scored in the 2012 season.

^b^ *,** significant at the 5% and 1% levels, respectively.

^c^ Spikelet length, spikelet width, and RDSA were scored by using SmilePlant.

**Figure 5:**
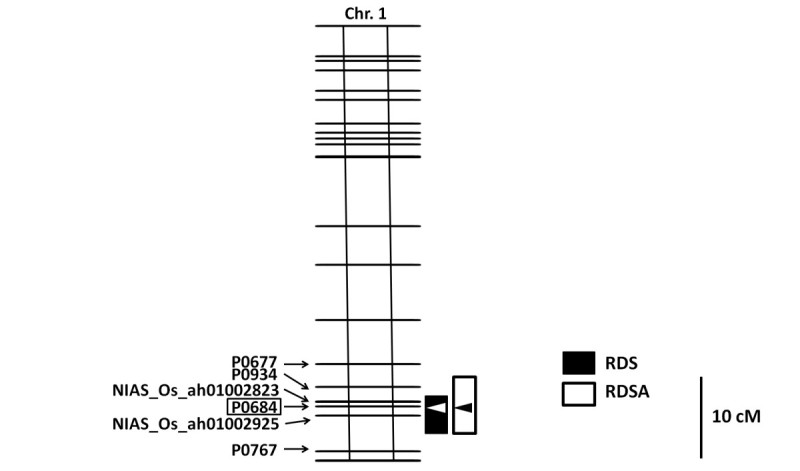
**Position of major QTL for BGR resistance on chromosome 1.** Marker names are shown on the left-hand side of the chromosome. Arrowheads and boxes represent LOD peaks of putative QTLs and their 1.5-LOD support intervals (Dupuis and Siegmund 1999), respectively. Black and white boxes indicate putative regions of QTLs for RDS and RDSA, respectively.

To genotype the BIL population, we used a 384-plex set of single-nucleotide polymorphism (SNP) markers that were selected by using diverse accessions of cultivated Asian rice (Ebana et al. 2010). Among the 384 markers, 245 detected polymorphism between Kele and Hitomebore (Additional file 4: Figure S2, Additional file 5: Table S3). One QTL, which was detected as having a major effect on both RDS and RDSA, was mapped near SNP marker P0684 on the long arm of chromosome 1 (Figure 5, Additional file 6: Figure S3). This QTL accounted for 25.7% of the total phenotypic variance in the BILs for RDS and 12.1% of the variance for RDSA (Table 2). The Kele allele decreased RDS and RDSA by 17.46% and 7.37%, respectively (Table 2). On the basis of the genotype at P0684, we classified each BIL as homozygous for either the Kele allele or the Hitomebore allele (Figure 4). BILs homozygous for the Kele allele at P0684 had RDS scores of 11.9% to 62.5% and RDSA scores of 5.5% to 36.1%. In contrast, RDS and RDSA in BILs homozygous for the Hitomebore allele ranged from 22.2% to 100% and from 10.8% to 71.0%, respectively. Thus, plants in the class homozygous for the Kele allele tended to show lower values for RDS and RDSA (indicating resistance) than those in the class homozygous for the Hitomebore allele. These results clearly demonstrate the existence of the QTL for BGR resistance on the long arm of chromosome 1. On the other hand, no QTLs for agronomic traits such as culm length, panicle length, number of panicles, spikelet length, spikelet width, or heading date were detected near P0684 (the marker linked to the QTL for RDS and RDSA) on the long arm of chromosome 1 (Additional file 7: Figure S4).

**Table 2:** **A putative QTL for BGR resistance (RDS and RDSA) detected in BILs derived from a cross between Kele and Hitomebore**

Traits	Chromosome	Nearest marker	cM^a^	LOD	***AE*** ^b^	***R*** ^2c^
Ratio of diseased spikelets (RDS)	1	P0684	42.1	7.0	17.46	25.7
Ratio of diseased spikelet area (RDSA)	1	P0684	42.1	3.3	7.37	12.1

^a^ Genetic distance from the end of the short arm of the chromosome to the QTL LOD peak.

^b^ Additive effect of the allele from Hitomebore compared with that from Kele. A positive number indicates that a higher disease score (RDS or RDSA) is associated with the allele from Hitomebore.

^c^ Percentage of phenotypic variance explained by each QTL.

## Discussion

### Identification of a major QTL for BGR resistance in rice

Genetic analysis of BGR is very difficult because the disease is highly influenced by environmental conditions (Tsushima 1996; Tsushima et al. 1985), and only one report of QTL analysis of BGR resistance (Pinson et al. 2010) has been published until now. Pinson et al. (2010) found a major QTL for BGR resistance co-located with a QTL for heading date. Because late-flowering panicles are subjected to cooler temperatures that are less conductive to disease development during grain fill, it is possible that the genetic effects of the heading-related QTLs affected the disease scoring. Pinson et al. (2010) concluded that further study of QTLs for diseases affected by heading date would be best pursed by using a disease evaluation method and/or genetic population not confounded by heading date variation.

By selecting parental cultivars with similar heading dates and using a method that minimizes the effect of heading date variation, we successfully detected a major QTL for BGR resistance on chromosome 1. No QTL for heading date was detected near the BGR resistance QTL on chromosome 1, indicating that the use of a cut-panicle method such as the modified method developed in this study enabled us to avoid the effects of heading date when screening for BGR resistance. We could not detect a QTL for either heading date or disease resistance near the one reported by Pinson et al. (2010) on chromosome 3.

In our experiments, some cultivars reported to be resistant when evaluated in the field (Prabhu and Bedendo 1988; Wasano and Okuda 1994) did not show BGR resistance when evaluated by using the cut-panicle method. The differences observed between our results and the studies reported previously could be a result of the use of different inoculation methods and growth conditions. Here, we analyzed the diseased spikelets 6 to 8 days after inoculation, whereas in field evaluations, plants are typically assessed 3 weeks after inoculation. Therefore, we are probably measuring the resistance to initial infection rather than resistance to bacterial spread in plants after infection. During maturation, RDS of Kele of 20 days after pot inoculation remained lower than that of Hitomebore, and the grain sterility rate of Kele of 30 days after pot inoculation was lower than that of Hitomebore (Additional file 2: Table S2). Therefore, it is possible that Kele is also resistant to BGR during grain filling process as well as initial infection. The major QTL identified on chromosome 1 could be responsible for initial infection and cut-panicle method may not be sensitive enough in detecting minor resistant QTLs. We are currently developing a near-isogenic line (NIL) for the QTL identified on chromosome 1 and are planning to conduct field evaluation of the NIL.

In this study, we slightly modified the screening method from that reported by Miyagawa and Kimura (1989). According to Tsushima et al. (1995), RDS was highest in spikelets that were inoculated exactly on the day of anthesis. RDS remained high when spikelets were inoculated within 3 days after anthesis, then decreased gradually beginning 4 days after anthesis. Spikelets inoculated before anthesis were reported to be resistant to BGR (Tsushima et al. 1995). These results were obtained from an analysis of *temperate japonica* cultivars such as Koshihikari, which are relatively susceptible to BGR, and we did not know whether the same pattern would be found in a more resistant cultivar. We tested whether Kele showed the same pattern of BGR resistance and found that Kele and Hitomebore showed different patterns of susceptibility to BGR resistance: the susceptibility of spikelets declined dramatically 1 day after anthesis in Kele but not in Hitomebore. Thus, the difference in RDS between Kele and Hitomebore is the greatest in spikelets inoculated 1 day after anthesis. For the above reasons, we modified the cut-panicle method by using only spikelets inoculated 1 day after anthesis for the QTL analysis. Based on the results obtained with the parental cultivars (Hitomebore and Kele), we expect that this modified method allowed us to detect differences in BGR resistance among the BILs with greater precision than the standard method. By using this method, we were able to detect a QTL for BGR resistance on chromosome 1.

The reason for the quick decline of susceptibility in Kele between anthesis and 1 day after anthesis is still unknown. One hypothesis is that the lemma and palea close more quickly in Kele than in Hitomebore, so that Kele is protected physically from the pathogen by 1 day after anthesis. Another hypothesis is that physiological factors such as accumulation of compounds related to resistance were induced in Kele shortly after anthesis.

### Progress toward improvement of BGR resistance in rice

We measured the panicle disease scores among 84 cultivars by the standard cut-panicle inoculation method and found a wide range of variation for BGR resistance among the *indica* and *tropical japonica* cultivars, whereas the *temperate japonica* cultivars were all susceptible (Figure 1B). Thus, it is necessary to introduce one or more genomic regions from *indica* or *tropical japonica* cultivars into *temperate japonica* in order to develop BGR-resistant cultivars of *temperate japonica*. To enable utilization of the QTL for BGR resistance detected in this study in breeding programs, we are currently developing a NIL for the QTL allele from Kele in the genetic background of Hitomebore (*temperate japonica*). Furthermore, additional resistance genes will be required to breed stable BGR-resistant cultivars. Thus, it is necessary to test resistant cultivars other than Kele to search for new QTLs.

## Conclusions

We mapped a QTL for BGR resistance on the long arm of chromosome 1 by using BILs derived from a cross between Kele and Hitomebore. The Kele allele at the QTL decreased both RDS and RDSA. By further analysis of the QTL, we could not only reveal the genetic mechanism of BGR resistance, but also advance the marker-assisted selection in rice breeding programs.

## Methods

### Plant materials

Eighty-four rice (*Oryza sativa* L.) cultivars were grown in paddy fields at NIAS in Tsukuba, Japan. Seeds from the WRC were obtained from the Gene Bank of the NIAS. WRC cultivars were categorized into *indica*, *temperate japonica* (Ebana et al. , and *tropical japonica*^2010^; Kojima et al. 2005). For the QTL analysis, 110 BILs were developed from BC_2_F_2_ plants produced by crossing Hitomebore (the recurrent parent) with Kele and were used to construct a linkage map and to search for QTLs for resistance to BGR.

### Assessment of panicle resistance to BGR

Eighty-four rice cultivars were grown in paddy fields in 2010. Thirty-day-old seedlings were transplanted at a density of one seedling per hill on 19 May. Each cultivar was planted in a single row of 10 hills at a spacing of 15 cm between hills and 30 cm between rows. Basal fertilizer was applied at a rate of 56 kg N, 56 kg P, and 56 kg K ha^-1^. Some cultivars exhibit extremely late heading under natural field conditions, so we used short-day (SD) equipment for those cultivars to promote heading. The SD equipment was set up in the field; the cover of the apparatus, which was able to shut out sunlight, automatically closed at 1700 hours and opened at 0800 hours. Cultivars were categorized by heading date and inoculated on different dates (from July 30 to August 23).

We measured resistance to bacterial grain rot in these cultivars by a cut-panicle inoculation method. This was based on the reported method for rice (Miyagawa and Kimura 1989), which was in turn based on the ‘cut-spike’ test developed for the evaluation of *Fusarium* head blight resistance in barley (Hori et al. 2005; Takeda and Heta 1989). Panicles at 3 days after heading were collected from the field, cut at the second internode from the top, and placed in a 500-ml Erlenmeyer flask of water. The bacterial strain (*Burkholderia glumae*), Kyu82-34-2 (MAFF 302744), which possessed stable virulence and was maintained at the National Institute of Agro-Environmental Sciences, was used as a source of inoculum. Bacterial inocula were incubated on agar-solidified LB medium (2% agar) at 28°C for 4 days and suspended in sterilized, distilled water at a concentration of 10^8^ per ml. Before inoculation, 0.01% Tween-20 was added to the suspension to increase adhesion of inocula on the panicles. Panicles were spray-inoculated with a freshly prepared inoculum suspension (approximately 1.7 to 2 ml per panicle) by a small sprayer. The inoculated panicles were then placed in a plant growth chamber maintained at 25°C and 100% humidity for 20 h. After the infection period, the panicles were covered with a transparent plastic bag and moved to another growth chamber maintained at 27°C and >80% relative humidity for 6 days under a 14-h photoperiod with light intensity of 1000 lux. Three panicles per cultivar were inoculated, and disease symptoms were scored 6 days after inoculation using a 0–10 scale, where 0 indicated 0% infected spikelets per panicle (resistant), and 10 indicated over 90% infected spikelets per panicle (susceptible).

Pot inoculation was conducted to confirm the BGR resistance of Kele and Hitomebore. We compared RDS of Kele and Hitomebore cultivated in the same condition between pot inoculation and standard cut-panicle inoculation at a fixed time from 3 to 5 days after heading. Seedlings of both cultivars were transplanted into soil (Bonsol No. 2, Sumitomo Kagaku Kougyo, Osaka, Japan) in 18-cm-diameter plastic pots. Plants were cultivated in a greenhouse at 25°C from November to April, and inoculation experiments were conducted in March. Bacterial inocula were prepared as described above. After inoculation, the pots were placed in a plant growth chamber maintained at 25°C and 100% humidity for 20 h. After the infection period, the inoculated plants were then returned to the greenhouse. For both of Kele and Hitomebore, eight plants (over ten panicles) were used for pot inoculation and at least six panicles were used for standard cut-panicle inoculation. Disease symptoms were scored 6 days after standard cut-panicle inoculation and disease symptoms were scored 6 days and 20 days after pot inoculation. Several types of spikelets were observed: (1) spikelets with no symptoms, (2) spikelets whose palea and lemma had reddish-brown lesions but diseased area <50% of spikelet area, and (3) spikelets with diseased area ≥50% (severe disease infection). Therefore, the RDS was determined as follows:

$$\mathrm{RDS}\left(\%\right)=\left(0.5N_{1}{+N}_{2}\right)100/\left(N_{0}+N_{1}+N_{2}\right)$$

N_0_ = number of spikelets with no symptoms

N_1_ = number of spikelets whose palea and lemma had reddish-brown lesions but diseased area <50% of spikelet area

N_2_ = number of spikelets with diseased area ≥50% (severe disease infection)

In order to compare the resistance to BGR between Kele and Hitomebore during grain filling process, the grain sterility rate of 30 days after pot inoculation was also scored. The each grain sterility rate of Kele and Hitomebore was compensated by the each sterility rate of spikelets without inoculation.

### Optimization of inoculation timing

In 2011, we compared the BGR susceptibility among spikelets at several time points before or shortly after anthesis. Methods of plant cultivation were essentially the same as in 2010. In most cultivars, anthesis starts in the morning and ends around early afternoon, and each spikelet is open for approximately 50 to 80 minutes (Matsuo and Hoshikawa 1993). Each morning at the paddy field, we selected panicles with large numbers of opening spikelets; removed all of the closed spikelets, so that the panicles would contain only spikelets that opened on that day; and labeled the selected panicles with the date of anthesis. For the cut-panicle inoculation, these prepared panicles were collected on the desired day after anthesis. The method of inoculation was the same as described above, and three panicles per line or cultivar were inoculated for each time point. Disease symptoms were scored 6 days after inoculation and used to calculate RDS.

### Evaluation of BILs

The BILs were grown in a paddy field at NIAS in 2012. Thirty-day-old seedlings were transplanted at one seedling per hill on 30 May. The methods used for plant cultivation were essentially the same as those in 2010 and 2011. Days to heading for Kele and Hitomebore were 80 and 82 days, respectively in 2012. In contrast, days to heading of the BILs ranged from 80 to 100 days. Therefore, the BILs were categorized by heading date and inoculated on different dates (from August 1 to August 23). Following the results from the inoculation timing experiments, we used a modified cut-panicle inoculation method in which panicles containing only spikelets at 1 day after anthesis were harvested and inoculated. Three panicles (ca. 40–60 spikelets) per each line were inoculated, and RDS and RDSA (described below) were scored 6 to 8 days after inoculation. The mean RDS and RDSA scores for each BIL were used for QTL analysis. Each inoculation test was confirmed by inclusion of Kele and Hitomebore as controls.

In the BIL study, three plants per line were measured for culm length, panicle length, number of panicles, and heading date. Heading date means days to heading from sowing. Three panicles (ca. 40–60 spikelets) per line were measured for spikelet length and width by using SmilePlant (described below).

### Image analysis

For the QTL analysis, we measured the RDSA as well as RDS. The inoculated spikelets were removed from the panicle and scanned with an EPSON GT-X820 scanner (Seiko Epson, Nagano, Japan). RDSA was calculated by SmilePlant (Software of Measuring Image for Lesion Estimation), which we developed for this analysis. SmilePlant was developed based on SmartGrain, high-throughput phenotyping software for measuring seed shape using image analysis (Tanabata et al. 2012). After being calibrated for the diseased color and normal color of the spikelets to be analyzed, SmilePlant can distinguish the diseased area from the normal area of each spikelet and calculate RDSA by measuring the number of pixels of diseased and normal areas. RDSA was calculated as ([number of pixels of diseased area/total number of pixels] × 100).

### DNA extraction and genotyping

Total DNA was extracted from leaves by the CTAB method (Murray and Thompson 1980), and 5 μl of 50 ng/μl DNA was used in the SNP analysis. We used a 384-plex set of SNP markers selected from diverse accessions of cultivated Asian rice (Ebana et al. 2010). Genotyping was performed by using the GoldenGate BeadArray technology platform (Illumina Inc., San Diego, CA, USA). These SNPs were detected by using the Illumina Bead Station 500G system. All experimental procedures for the SNP typing followed the manufacturer’s instructions.

### Statistical and QTL analyses

We performed linkage mapping using version 3.0 of MAPMAKER/EXP (Lander et al. 1987), and we used the Kosambi map function to calculate genetic distances. We performed QTL analyses by using composite interval mapping, as implemented by the Zmapqtl program (model 6) provided in version 2.5 of the QTL Cartographer software (Wang et al. 2005). We used genome-wide threshold values (α = 0.05) to detect putative QTLs on the basis of the results of 1000 permutations. To compare the resistance between Kele and Hitomebore, we used the Student’s *t* test provided by JMP version 9.0 software (SAS Institute, Cary, NC, USA).

## Electronic supplementary material

Additional file 1: Table S1: Cultivars screened for BGR resistance. (XLS 20 KB)

Additional file 2: Table S2: Comparison of BGR resistance between Kele and Hitomebore. (XLS 8 KB)

Additional file 3: Figure S1: Frequency distributions of agronomic traits of BILs derived from a cross between Kele and Hitomebore. White and black arrowheads indicate mean values for Hitomebore and Kele, respectively. (TIFF 193 KB)

Additional file 4: Figure S2: Linkage map for BILs derived from a cross between Kele and Hitomebore. The numbers on the left-hand side of each chromosome indicate map distances (cM) obtained using the Kosambi function. SNP marker names are shown on the right-hand side of each chromosome. Marker names are presented as abbreviations, which are defined in Additional file 2: Table S2. chr, chromosome number. (TIFF 2 MB)

Additional file 5: Table S3: List of SNP markers used in the QTL analysis of BILs derived from a cross between Kele and Hitomebore. The SNP markers were selected from genome-wide SNP marker data (Ebana et al. 2010). (XLS 28 KB)

Additional file 6: Figure S3: LOD scans from QTL analysis of ratio of diseased spikelets (RDS) and ratio of diseased spikelet area (RDSA), measured by the modified method of cut-panicle inoculation, in BILs derived from a cross between Kele and Hitomebore. LOD score profiles of each chromosome (from chromosome Ch1 to Ch12) are oriented with the short arm of each chromosome at the left. Blue LOD curve indicates RDS; red LOD curve indicates RDSA. The LOD threshold used to declare putative QTLs for RDS and RDSA is indicated by the horizontal lines. (TIFF 194 KB)

Additional file 7: Figure S4: Log-likelihood (top) and additive-effect plots (bottom) across (A) all 12 rice chromosomes and (B) chromosome 1 from the QTL analyses for BGR resistance (RDS and RDSA) and agronomic traits in BILs derived from a cross between Kele and Hitomebore. The LOD threshold used to declare putative QTLs in each population is indicated by the horizontal line. RDS: ratio of diseased spikelets, RDSA: ratio of diseased spikelet area, CL: culm length, PL: panicle length, NP: number of panicles, SL: spikelet length, SW: spikelet width, HD: heading date. (TIFF 2 MB)

Below are the links to the authors’ original submitted files for images.

Authors’ original file for figure 1

Authors’ original file for figure 2

Authors’ original file for figure 3

Authors’ original file for figure 4

Authors’ original file for figure 5
