# Supplementary figures and images for: Mapping a quantitative trait locus for resistance to bacterial grain rot in rice
Source: Rice (N Y). 2013 May 21;6:13. doi: 10.1186/1939-8433-6-13 (PMC4883728; doi:10.1186/1939-8433-6-13)

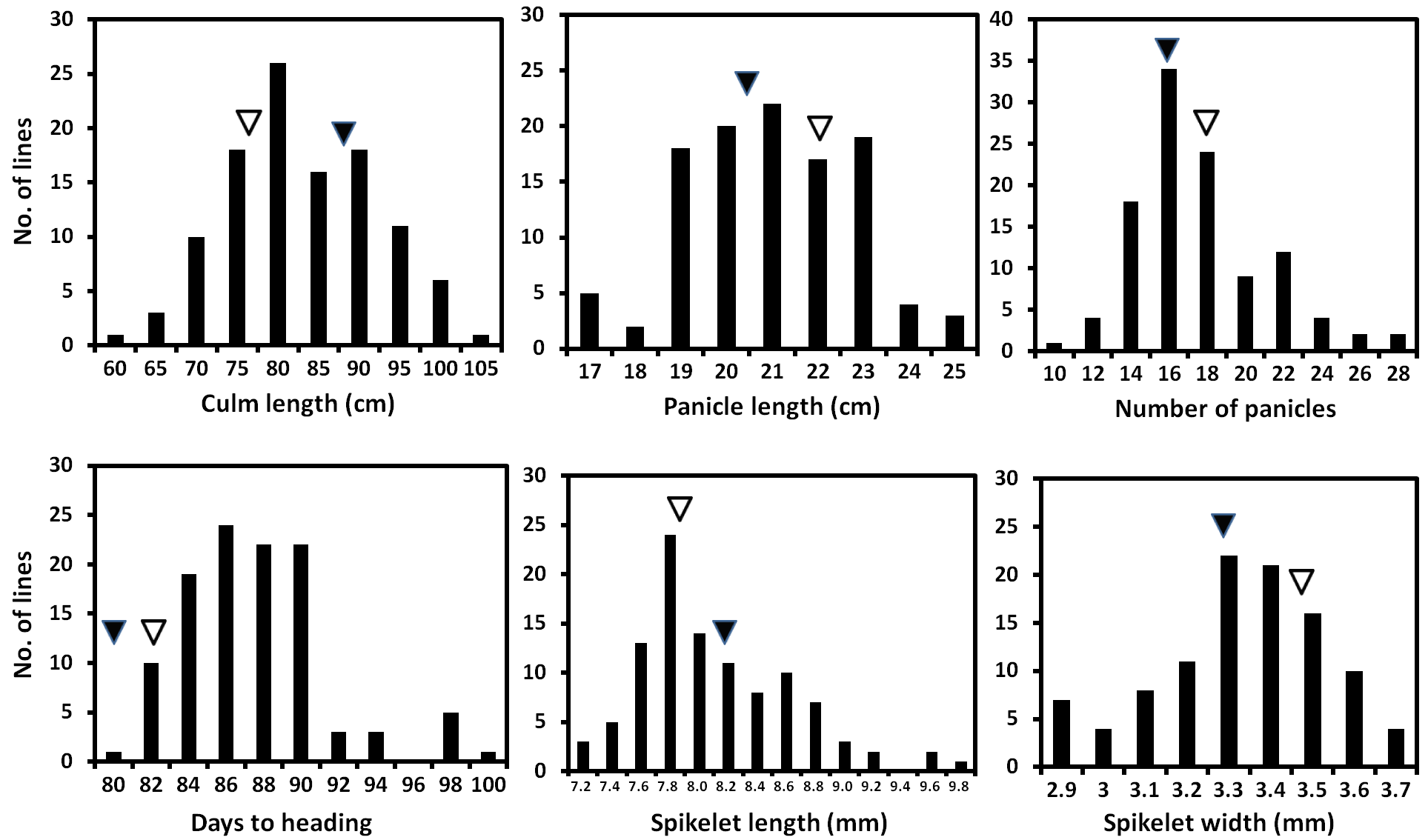

Supplement: Supplementary file 3 — Additional file 3: Figure S1: Frequency distributions of agronomic traits of BILs derived from a cross between Kele and Hitomebore. White and black arrowheads indicate mean values for Hitomebore and Kele, respectively. (TIFF 193 KB) [file 12284_2012_49_MOESM3_ESM.tiff]

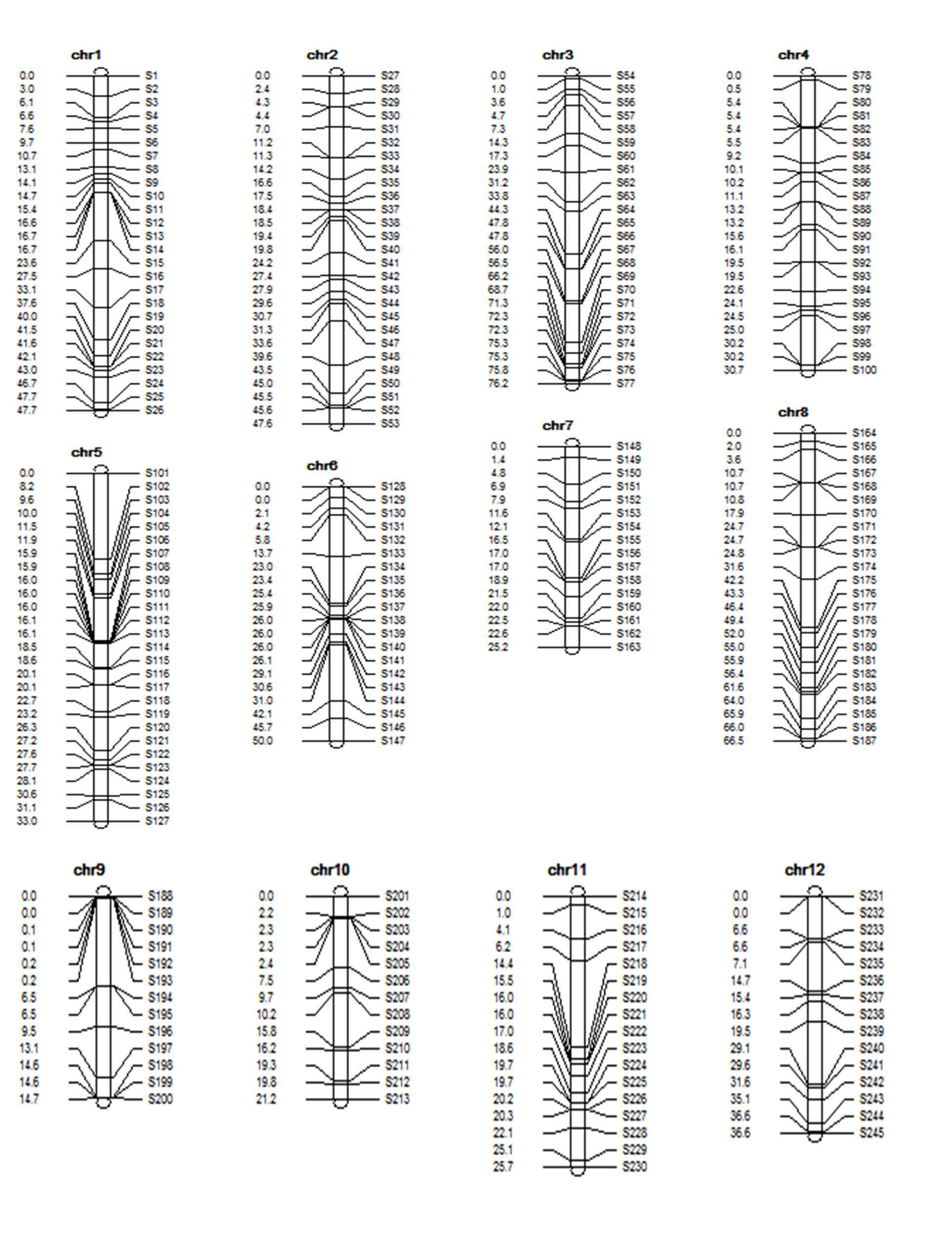

Supplement: Supplementary file 4 — Additional file 4: Figure S2: Linkage map for BILs derived from a cross between Kele and Hitomebore. The numbers on the left-hand side of each chromosome indicate map distances (cM) obtained using the Kosambi function. SNP marker names are shown on the right-hand side of each chromosome. Marker names are presented as abbreviations, which are defined in Additional file 2: Table S2. chr, chromosome number. (TIFF 2 MB) [file 12284_2012_49_MOESM4_ESM.tiff]

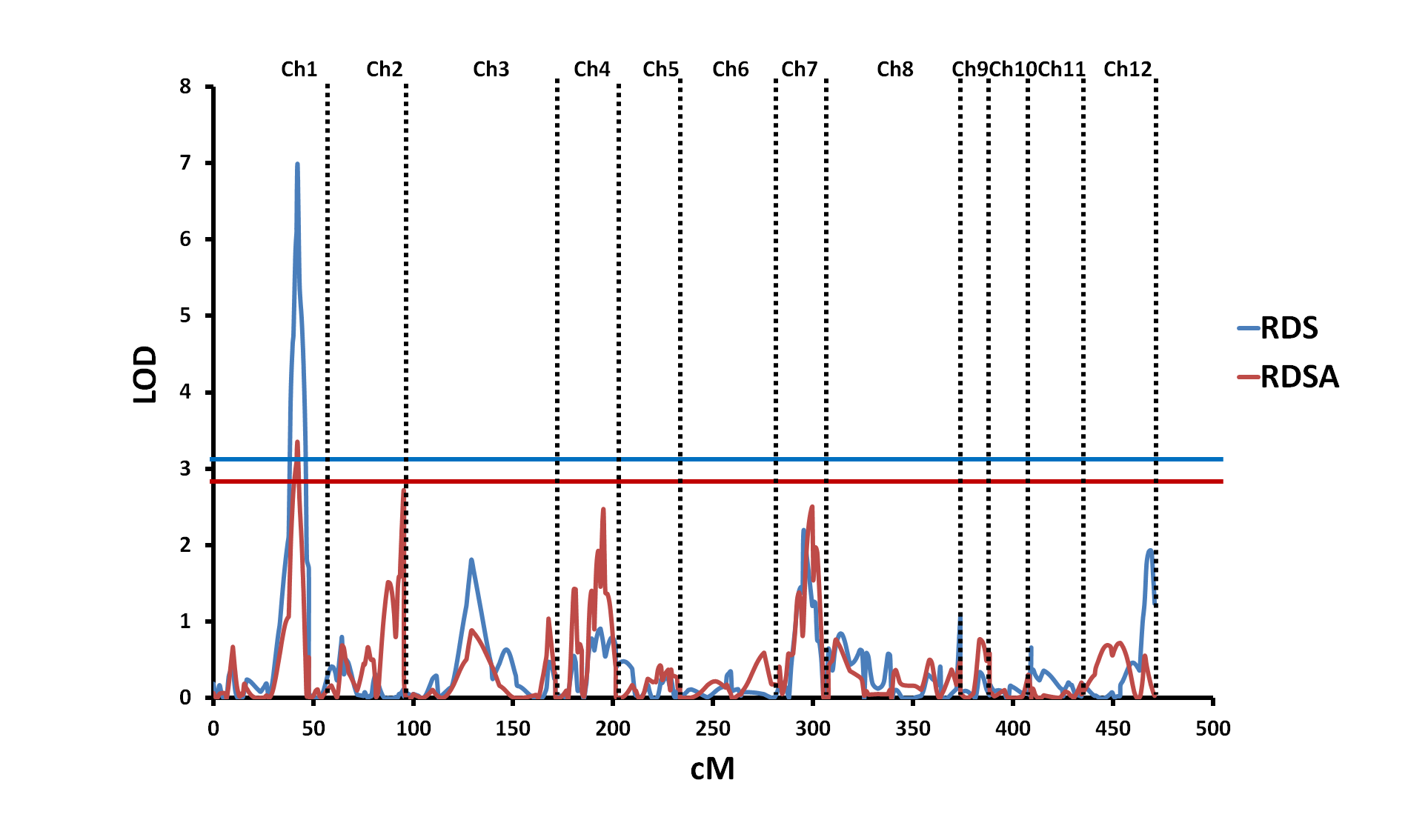

Supplement: Supplementary file 6 — Additional file 6: Figure S3: LOD scans from QTL analysis of ratio of diseased spikelets (RDS) and ratio of diseased spikelet area (RDSA), measured by the modified method of cut-panicle inoculation, in BILs derived from a cross between Kele and Hitomebore. LOD score profiles of each chromosome (from chromosome Ch1 to Ch12) are oriented with the short arm of each chromosome at the left. Blue LOD curve indicates RDS; red LOD curve indicates RDSA. The LOD threshold used to declare putative QTLs for RDS and RDSA is indicated by the horizontal lines. (TIFF 194 KB) [file 12284_2012_49_MOESM6_ESM.tiff]

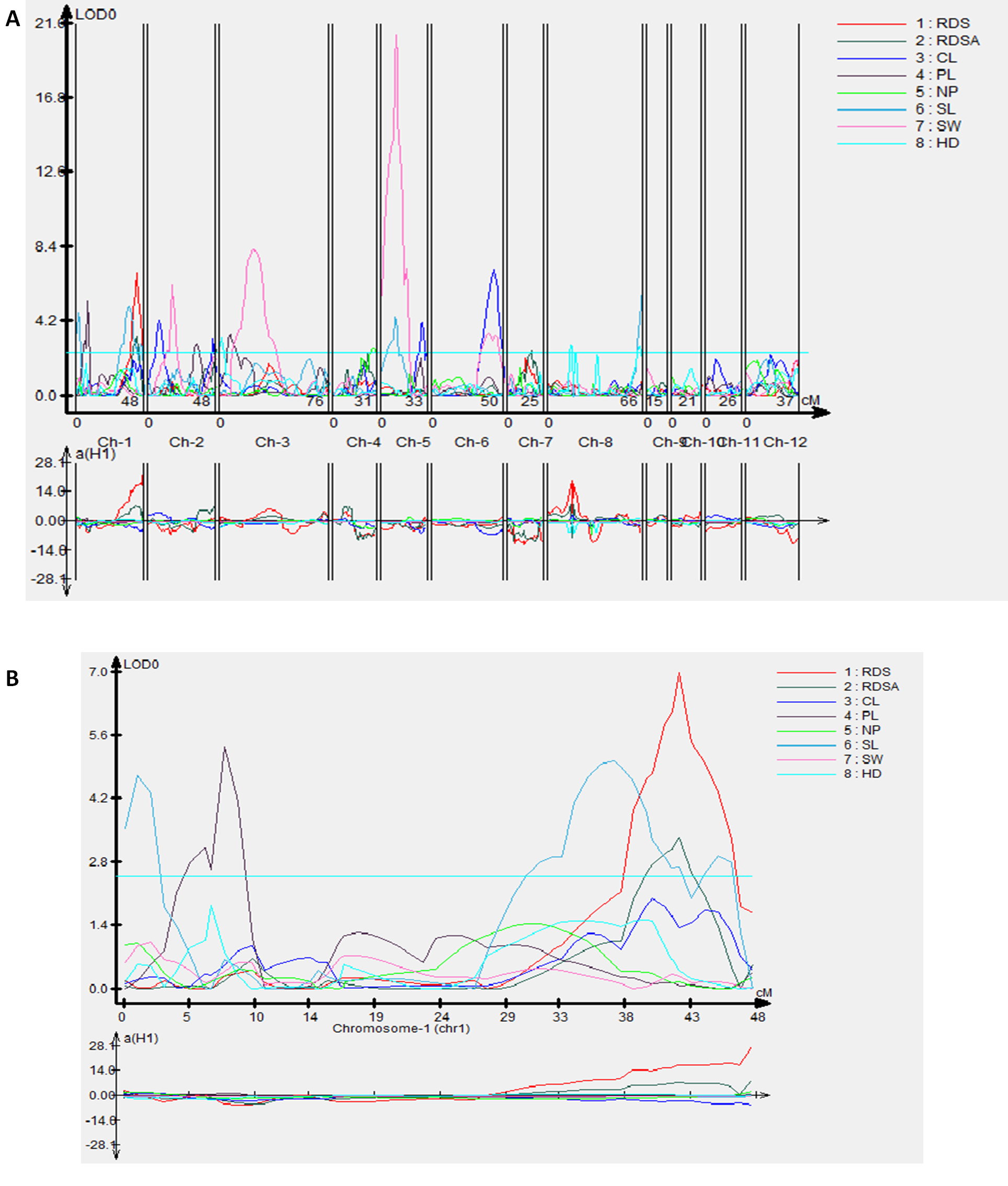

Supplement: Supplementary file 7 — Additional file 7: Figure S4: Log-likelihood (top) and additive-effect plots (bottom) across (A) all 12 rice chromosomes and (B) chromosome 1 from the QTL analyses for BGR resistance (RDS and RDSA) and agronomic traits in BILs derived from a cross between Kele and Hitomebore. The LOD threshold used to declare putative QTLs in each population is indicated by the horizontal line. RDS: ratio of diseased spikelets, RDSA: ratio of diseased spikelet area, CL: culm length, PL: panicle length, NP: number of panicles, SL: spikelet length, SW: spikelet width, HD: heading date. (TIFF 2 MB) [file 12284_2012_49_MOESM7_ESM.tiff]

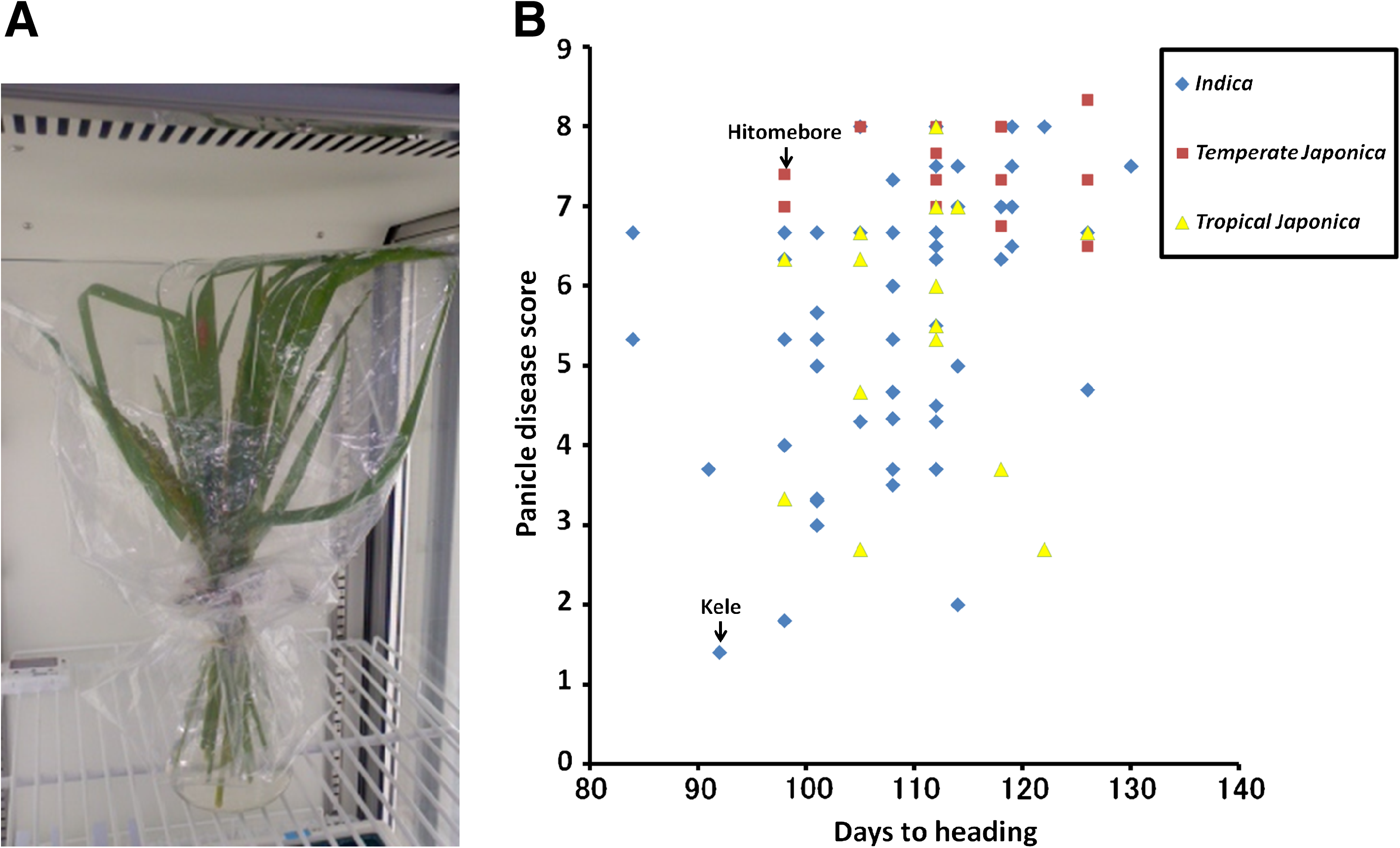

Supplement: Supplementary file 8 — Authors’ original file for figure 1 [file 12284_2012_49_MOESM8_ESM.tif]

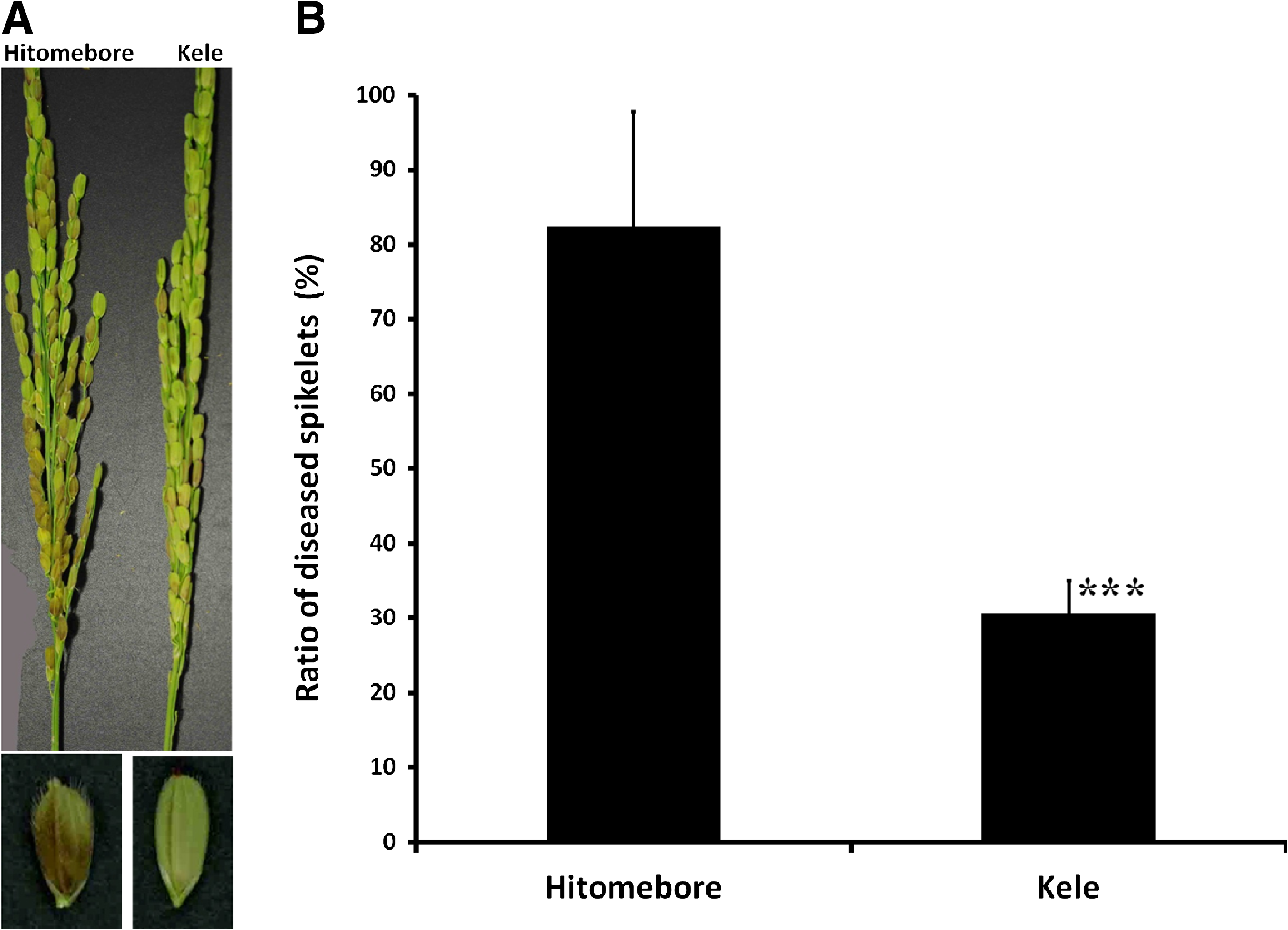

Supplement: Supplementary file 9 — Authors’ original file for figure 2 [file 12284_2012_49_MOESM9_ESM.tif]

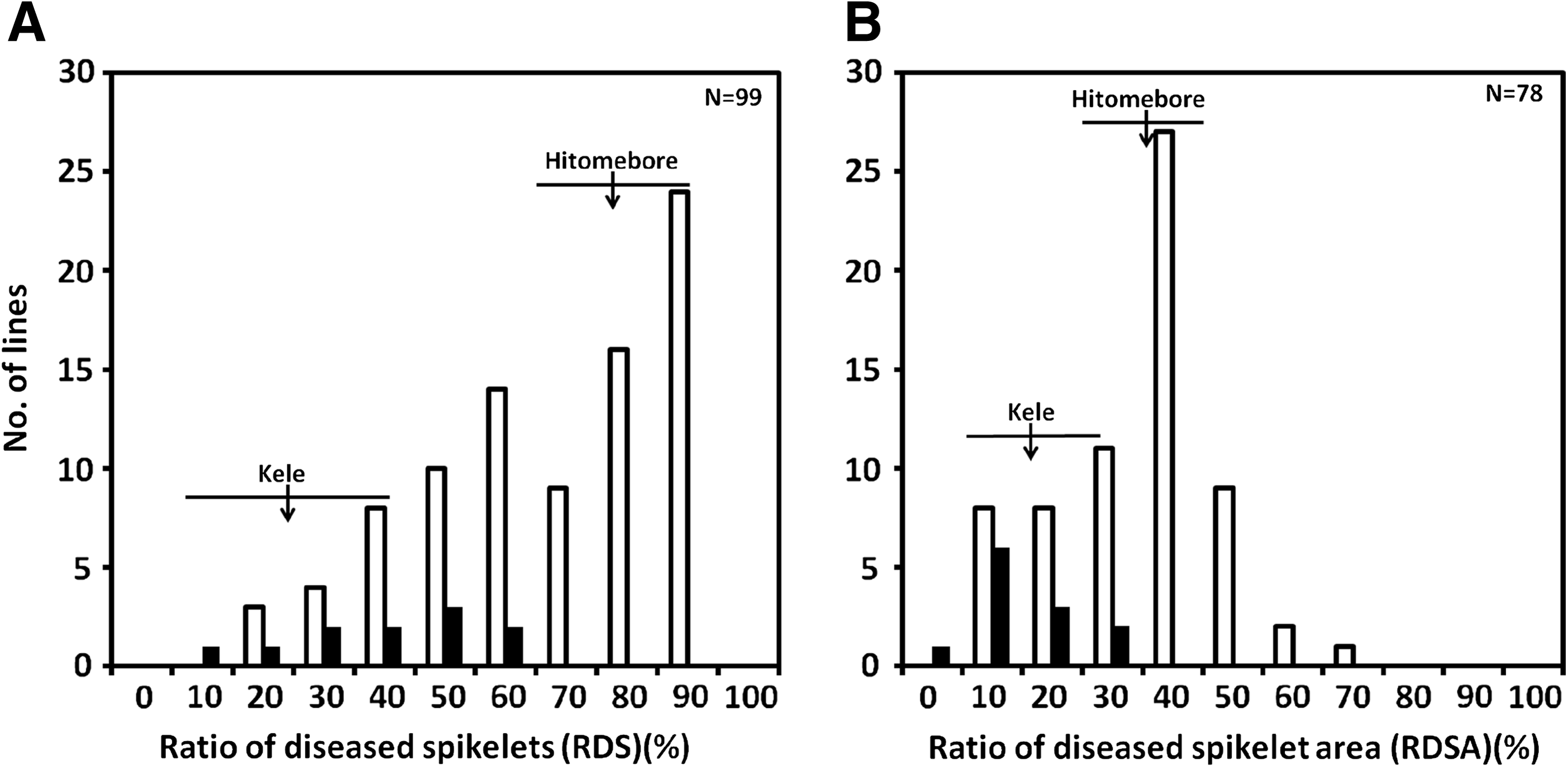

Supplement: Supplementary file 11 — Authors’ original file for figure 4 [file 12284_2012_49_MOESM11_ESM.tif]

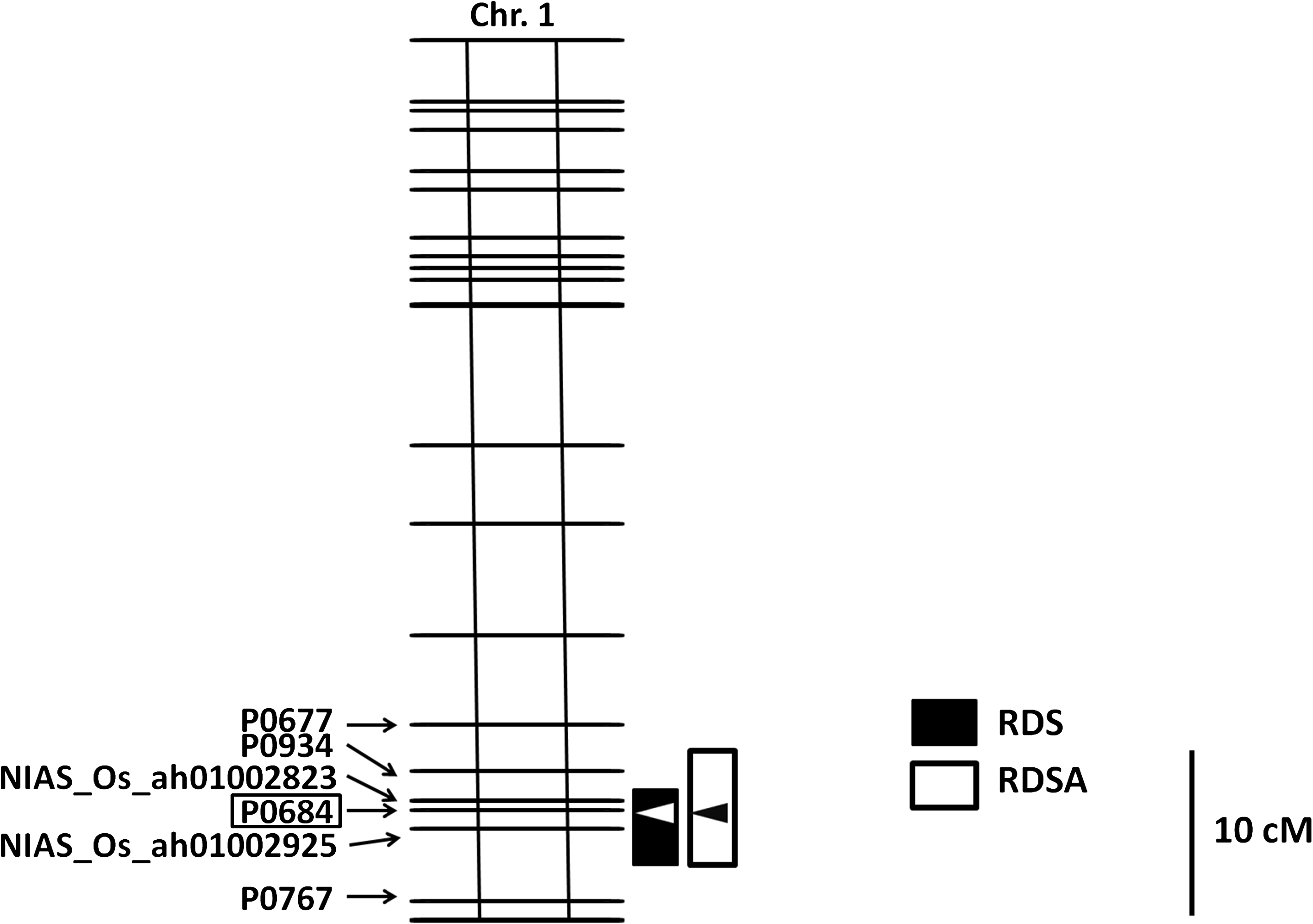

Supplement: Supplementary file 12 — Authors’ original file for figure 5 [file 12284_2012_49_MOESM12_ESM.tif]
